# The crisis we are not naming: The psychology of capitalism

**DOI:** 10.1111/bjso.70044

**Published:** 2026-01-19

**Authors:** Karim Bettache

**Affiliations:** ^1^ The Chinese University of Hong Kong Shatin, N.T. Hong Kong

**Keywords:** capitalism, competitiveness, cultural psychology, cultural syndromes, entrepreneurialism, individualism, self‐optimization

## Abstract

Psychology has rendered capitalism invisible, treating individualism as cultural inheritance rather than a response to contemporary economic conditions. Building on my recent theoretical framework (Bettache, *Personality and Social Psychology Review*, 29, 215, 2024), this article explores how capitalism functions as a missing link in psychology—an overlooked generative mechanism that shapes the phenomena we study. Three ‘capitalist syndromes’—Gain Primacy Syndrome (perpetual accumulation as life orientation), Zero‐Sum Rivalry Syndrome (competitive ethos eroding social bonds) and Ownership Syndrome (possessive identity formation)—interact recursively to generate a self‐enhancement agenda we recognize as individualism. This framework reinterprets established findings, from the correlation between economic development and individualism to social class differences in self‐concept, as responses to political‐economic structures rather than ancient traditions. Making capitalism visible transforms psychological distress from individual pathology into rational responses to structural dysfunction, opening possibilities for interventions that address root causes rather than merely helping individuals cope with harmful conditions.

Across the world, we face converging crises that seem disconnected but share common roots. Anxiety and depression rates have surged, particularly among young people, with university students reporting unprecedented levels of stress and burnout (Haruna et al., 2025; Macalli et al., 2025). Social isolation has reached epidemic proportions, with people reporting fewer close friendships and weaker community bonds than previous generations (Holt‐Lunstad, 2023, 2024). Educational systems generate intense pressure and competition while leaving students feeling that learning serves primarily to boost future earning potential (Becker & Börnert‐Ringleb, 2025; Navarro et al., 2025). Workers experience mounting job insecurity, longer hours and constant demands for self‐improvement and ‘upskilling’ just to maintain their positions (Ballard et al., 2025; Canady, 2025).

The common response treats these as separate problems requiring separate solutions: mental health apps for anxiety, social skills training for loneliness, resilience programs for stressed students, mindfulness courses for burned‐out workers. Yet this fragmented approach overlooks a fundamental question: What if these aren't isolated issues but symptoms of a deeper structural problem? What if the economic system organizing our societies—*capitalism*—shapes our psychology in ways that generate these crises? And what if many of our proposed solutions—products to be purchased, programs to be sold, apps to be downloaded—operate within the very logic that created the problems?

Psychology has a capitalism problem—not in the sense that it actively promotes capitalism, but in that it renders capitalism invisible.^1^ We study individualism without acknowledging the economic system that rewards individual competition. We examine materialism without questioning the profit‐driven industries that manufacture desire. We document self‐enhancement biases without recognizing the market pressures that make constant self‐optimization a survival strategy. This omission is not merely an oversight—it represents a fundamental gap in how we understand culture itself.

When researchers document that university students are intensely individualistic and competitive, we attribute this to ‘Western culture’ (e.g., Ma & Schoeneman, 1997). When we observe widespread materialism and consumption‐driven identity formation, we label it a side‐effect of ‘WEIRD’ societies—Western, Educated, Industrialized, Rich, Democratic. But the traditional cultural attribution obscures more than it reveals. It treats these psychological patterns as deep‐rooted cultural inheritances—something about ‘the West’ going back centuries (Greek philosophy, Protestant theology and Enlightenment thought)—rather than as responses to contemporary economic conditions. A student's anxiety about class rankings perhaps is not an echo of ancient Greek individualism; it may be a response to an educational system restructured around market competition. A worker's compulsion to self‐optimize might not reflect a timeless Western trait; it could be adaptation to labour markets that demand constant productivity gains. The materialism we observe may not be cultural destiny; it might be cultivated by profit‐driven industries that manufacture desire. By rendering capitalism invisible, psychology fails to grasp a primary force shaping culture, social relations and human wellbeing in the modern world.

Understanding how capitalism becomes invisible requires attending to the psychological processes through which any cultural system becomes naturalized—rendered inevitable, universal or simply ‘how things are’ rather than a contingent historical arrangement (Bettache, 2024; Gergen, 1973; Malherbe, 2025; Sullivan, 2020). Capitalism saturates our cultural environment so completely that it becomes the water we swim in, unnoticed precisely because it is omnipresent. When every institution—schools, hospitals, governments and families—operates according to market logic, that logic ceases to appear as one possible organizing principle among many and instead appears as common sense.

This saturation shapes not only institutions but also the interpretive resources available for making sense of ourselves. Capitalism provides the cultural affordances—the scripts, schemas and ready‐made frameworks—through which we understand our social world (Adams et al., 2019; Goddard & Wierzbicka, 2004). When a student experiences anxiety about grades, the available cultural scripts interpret this as individual performance anxiety requiring individual solutions: study harder, manage stress better, build resilience. The script that might frame this anxiety as a rational response to an educational system restructured around market competition remains culturally unavailable—not because it is wrong, but because capitalism does not provide affordances for its own critique.

Psychology as a discipline has participated in this naturalization by treating capitalist‐produced psychological patterns as universal features of human nature or as cultural inheritances from ancient civilizations. When we label competitive individualism as ‘Western culture’ rooted in Greek philosophy, we obscure its contemporary economic foundations and render it resistant to change. After all, one cannot easily modify ancient cultural traditions, but one can reform economic policies and institutional structures. The naturalization of capitalism thus serves to foreclose possibilities for alternatives.

I recently proposed that capitalism functions as a missing link in much of psychology; the overlooked generative mechanism that can help explain cultural patterns we have long observed but incompletely understood (Bettache, 2024). By integrating capitalism into our theoretical frameworks, we can forge a new synthesis that connects individual psychology, cultural values and political‐economic structures into a coherent whole. This synthesis offers not just better explanations of existing phenomena, but transformative insights into how culture operates and evolves in the contemporary world.

## BEYOND GEOGRAPHY: THE ECONOMIC FOUNDATIONS OF CULTURE

Cultural psychology has made tremendous strides in documenting psychological differences across societies. We know that people in Western nations tend toward individualism while those in East Asian nations lean toward collectivism (Hofstede, 1984; Markus & Kitayama, 1991). We understand that subsistence patterns—rice farming versus wheat farming, herding versus fishing—shape cognitive styles and social orientations (Talhelm et al., 2014; Uskul et al., 2008). We recognize that historical factors like religious traditions and philosophical systems leave lasting cultural imprints (Nisbett, 2004).

Yet these explanations, while valuable, often treat economic systems as mere background conditions rather than active cultural forces. Consider a puzzle: Why do highly educated Kenyans exhibit Western‐style independent self‐concepts while tribal Kenyans maintain interdependent self‐concepts? Ma and Schoeneman (1997) studied this question and concluded that ‘Westernization’ through education likely explained this effect. But this merely labels the phenomenon rather than explaining it. What specifically about Western education produces this shift?

The answer becomes clear when we examine Kenya's economic trajectory. During the 1970s–1990s, Kenya underwent massive economic restructuring—trade liberalization, privatization of state industries and educational reforms explicitly designed to produce graduates aligned with global market demands (Mukhwana et al., 2017; Page, 2012). Higher education became a gateway to participation in capitalist economic networks, with attendant pressures toward competitive individualism, entrepreneurial self‐concepts and market‐oriented values. The ‘Western’ self‐concept is perhaps, more precisely, a *capitalist* self‐concept.

This reframing suggests a provocative hypothesis: many cultural differences attributed to ancient traditions or geographical factors may actually reflect varying degrees of capitalist economic integration. The dimension we call individualism–collectivism may be substantially shaped by how deeply capitalist principles penetrate a society's institutions, labour markets and reward structures.

## CAPITALISM AS CULTURAL GENERATOR: A NEW FRAMEWORK

Capitalism is more than an economic system; it is a cultural architect that constructs the very scaffolding of our psychological lives. At its core, capitalism operates through at least three fundamental principles: the profit motive (the drive for continuous capital accumulation and economic growth), market competition (the imperative for actors to outcompete rivals for market advantages) and private property ownership (the legal and cultural framework that enables exclusive control over productive assets and personal possessions). Through what I term ‘capitalist syndromes’—coherent patterns of beliefs, values and behaviours organized around these core principles—this economic system infiltrates our consciousness in ways that extend far beyond the marketplace (Bettache, 2024).

### The pressure for perpetual gain

Capitalism's profit motive—the imperative for constant growth and accumulation—does not stay confined to businesses. It seeps into how we think about ourselves and our lives. I term this the Gain Primacy Syndrome. When education systems increasingly frame learning as investment in human capital (Becker, 1993), when career‐success metrics revolve around income and net worth, when people internalize entrepreneurial identities that view the self as a brand to be optimized (Bröckling, 2015), we witness the cultural elevation of perpetual accumulation. This is not simply materialism—it is a comprehensive reorientation of life goals toward gain maximization.

How does the profit motive migrate from boardrooms into bedrooms, from corporate balance sheets into personal life goals? The answer lies in cultural affordances—the interpretive resources, behavioural scripts and evaluative frameworks that a cultural context makes readily available (Adams et al., 2019; Bettache, 2023, 2024). In societies organized around capitalist principles, the language of investment, returns and optimization becomes the default vocabulary for understanding virtually any domain of life (Goddard & Wierzbicka, 2004). We speak of ‘investing’ in relationships, calculating the ‘return’ on educational credentials, ‘optimizing’ our health and productivity, building our personal ‘brand’. These are not merely metaphors—they are cognitive tools that shape how we perceive choices and evaluate outcomes. When a culture's dominant institutions reward accumulation and growth, people draw upon these accumulation‐oriented scripts to navigate domains far removed from formal economic activity. A person deciding whether to maintain a friendship may unconsciously calculate whether the relationship offers sufficient ‘value’. A parent considering how to spend time with children may frame it as ‘quality time’—a formulation that imports productivity logic into intimate relations. The profit motive seeps into non‐economic domains not through explicit ideological instruction, but through the cultural affordances capitalism makes most cognitively available and socially rewarded (Bettache, 2024; Goddard & Wierzbicka, 2004).

The psychological costs are substantial. When self‐worth becomes tied to economic productivity and visible success, people experience constant pressure for self‐improvement. Research on ‘entrepreneurial selves’ reveals elevated stress, anxiety and burnout as individuals struggle to meet endless demands for optimization (Bröckling, 2015; Cardon & Patel, 2015). University faculty report that academic work has transformed from intellectual pursuit to revenue generation, fundamentally changing how they experience their profession (Mendoza et al., 2012). Even creative fields become subordinated to market logic, with artists and writers measuring success primarily through sales rather than cultural contribution (Mylonas, 2012).

### The corrosive effects of competition

Market competition requires economic actors to vie for market share, investment capital and consumer attention. But competition does not remain confined to markets—it infiltrates education through ranking systems and standardized testing, workplaces through performance metrics and forced distributions, and social life through status competition and conspicuous consumption (Hursh, 2007). I term this the Zero‐Sum Rivalry Syndrome. The competitive ethos cultivated by capitalist markets breeds adversarial tendencies that profoundly impact social relations and individual psychology.

The psychological research on competition is clear and troubling. Competitive environments consistently increase negative emotions, reduce interpersonal trust and erode the social bonds necessary for wellbeing compared to cooperative settings (Colpaert et al., 2015; Sherman, 1986). People in more market‐oriented societies exhibit stronger ‘zero‐sum’ beliefs—viewing others' gains as threatening their own prospects—which undermines empathy and mutual support (Różycka‐Tran et al., 2015). Perhaps most strikingly, the competitive pursuit of financial success correlates with poorer relationship quality, reduced wellbeing and increased risk of mental health problems (Kasser & Ryan, 1993; Sheldon et al., 2000).

The intensity of these effects varies with how deeply market competition penetrates a society. Japan's more regulated capitalism produces less antagonistic competitive orientations than Hong Kong's more laissez‐faire system, despite both societies sharing Confucian heritage (Dalgaard, 1993). Also, mainland China's recent marketization has measurably eroded interpersonal trust at the psychological level (Zhang & Xin, 2019). These patterns suggest that competitive psychology is not fixed but responds to economic policy choice.

### The trap of possessive identity

Capitalism centres private ownership as the foundation of economic organization and personal freedom. This principle extends beyond productive assets to consumer goods, with ownership becoming tightly intertwined with identity. I term this the Ownership Syndrome. In capitalist societies, people show enhanced memory for owned objects and brain activation in self‐referential regions when contemplating possessions (Kim & Johnson, 2015). Materialism—the tendency to value material possessions and wealth as central to one's identity, life goals and definitions of success—becomes a dominant mode of self‐presentation, particularly on social media platforms designed to monetize conspicuous consumption (Kasser, 2003; Pellegrino et al., 2022).

The costs of materialistic orientations to wellbeing are well‐documented. People who strongly prioritize material success and possessions exhibit lower self‐esteem, increased anxiety and depression, higher rates of substance abuse, and reduced life satisfaction compared to those valuing intrinsic goals like personal growth and relationships (Dittmar et al., 2014; Kasser & Ryan, 1993). Materialism also undermines social relationships—people with stronger materialistic values engage in less cooperation, show reduced concern in close relationships, and are less likely to help others (Sheldon et al., 2000; Sheldon & Kasser, 1995).

Critically, materialistic values do not emerge randomly. They are cultivated through profit‐driven advertising industries that deliberately manufacture insecurity and desire, social comparison mechanisms amplified by social media, and economic conditions that make material success the primary recognized marker of achievement (e.g., Dittmar, 2007). Consider the automobile industry, which sells not merely transportation but identity. Luxury car advertisements depict vehicles as emblems of elevated status and self‐actualization, associating ownership with success, freedom or sophistication depending on the target demographic (Elliott & Wattanasuwan, 1998). Similarly, high‐end fashion brands cultivate an aura of exclusivity around their products, transforming garments from functional items into identity markers—consumers pay premium prices not for superior materials but for logos that communicate belonging to particular social tribes, whether luxury fashion houses signaling wealth or streetwear brands signaling cultural authenticity (Rocamora, 2002; Vohra, 2016). Social media platforms amplify these dynamics through algorithmic curation that maximizes engagement by promoting images of others' possessions precisely calibrated to generate envy and inadequacy (Pellegrino et al., 2022). The result is a cultural environment where self‐worth becomes entangled with what one owns and displays. Understanding materialism as a response to these manufactured conditions, rather than as a personality flaw or cultural inheritance, fundamentally changes how we might address it.

## THE INDIVIDUALIST SYNTHESIS

These three syndromes do not operate independently. I argue that they interact recursively to generate a self‐enhancement agenda—the overarching pattern we recognize as individualism. The profit motive establishes wealth accumulation as a primary life goal. Market competition frames social relations as rivalrous rather than cooperative. Private ownership ties self‐worth to material acquisitions. Together, these dynamics produce the independent self‐construal, self‐optimization orientation and dispositional thinking patterns characteristic of individualist cultures (Hamamura et al., 2018; Varnum & Grossmann, 2017).

This synthesis offers fresh explanations for established findings. Consider the robust correlation between economic development and individualism (Gorodnichenko & Roland, 2011). Rather than viewing this as a byproduct of modernization per se, we can understand it as reflecting capitalism's expansion into new domains—the marketization of formerly non‐commercial spheres, the privatization of public goods and the intensification of competitive labour markets. As capitalism deepens its reach, individualist orientations strengthen correspondingly.

The framework also illuminates cultural dynamics. Why does socioeconomic status predict self‐concept across cultures, with higher‐SES individuals exhibiting more independent orientations (Kraus et al., 2011; Stephens et al., 2007)? Because class position determines exposure to capitalist imperatives—wealthier individuals participate more directly in capital accumulation, market competition and property ownership. The ‘culture of poverty’ versus ‘culture of affluence’ may reflect differential integration into capitalist economic structures.

Even historical cultural shifts become interpretable through this lens. Japan's rapid individualization over recent decades (Hamamura, 2012) coincides with neoliberal economic reforms intensifying market competition and privatization (Taniguchi & Kaufman, 2019). China's movement toward greater individualism (Steele & Lynch, 2013) tracks its embrace of market mechanisms under ‘socialism with Chinese characteristics’. These are not random drifts or inevitable stages of development—they are responses to changing political‐economic conditions.

## FROM UNDERSTANDING TO ACTION

Recognizing capitalism's psychological impacts opens up new possibilities for intervention—not just helping individuals cope with harmful conditions, but changing those conditions themselves. These interventions span education, work and mental health practice.

In education, current policies increasingly subordinate learning to market logic through standardized testing, rankings and ‘human capital’ framing (Au, 2016; Natale et al., 2015). Yet evidence points toward alternatives. Cooperative learning environments produce equal or better academic outcomes while significantly improving student wellbeing, reducing anxiety and fostering stronger social bonds compared to competitive classrooms (Sherman, 1986; Wheeler & Ryan, 1973). Moving away from competitive grading curves and class rankings toward competency‐based assessment focused on mastery rather than relative performance could preserve educational rigour while reducing psychological harm. Equally important is diversifying how we measure educational success beyond future earning potential to include civic engagement, creative expression and personal development—treating education as intrinsically valuable rather than merely instrumental to career advancement (Kasser & Ryan, 1996; Nussbaum, 2009).

In the workplace, environments structured around individual competition and performance metrics generate psychological distress while often failing to improve actual productivity (Bal & Dóci, 2018; Pignata & Winefield, 2015). Alternative organizational models offer evidence that different structures produce different psychologies. Worker cooperatives and employee ownership structures align individual and collective interests in ways that conventional hierarchical firms do not. In the United States, employee stock ownership plans (ESOPs) now cover millions of workers, and research finds that employee‐owners report greater organizational commitment, higher job satisfaction and lower intention to leave compared to employees in conventional firms (Blasi et al., 2018). These examples suggest that the competitive, zero‐sum psychology documented in conventional workplaces is not an inevitable feature of economic organization but responds to specific ownership and governance structures.

In clinical practice, capitalism‐conscious approaches are emerging that explicitly connect individual distress to structural conditions. The Liberation Psychology movement, drawing on the work of Ignacio Martín‐Baró, has developed frameworks for helping clients distinguish between problems requiring personal change and those requiring collective action (Malherbe, 2025). Community mental health models in Latin America and community psychology approaches in South Africa have pioneered interventions that address structural determinants of psychological distress alongside individual support, while critical psychology networks in the United Kingdom and United States have developed resources for clinicians seeking to contextualize client distress within political‐economic conditions (Adams et al., 2019; Bettache & Chiu, 2019). A capitalism‐aware clinical approach means helping clients recognize when their distress signals not personal inadequacy but structural dysfunction. When a graduate student experiences anxiety about academic competition, or a worker suffers burnout from impossible productivity demands, these may be rational responses to unreasonable conditions rather than individual pathology requiring medication or resilience training (Bettache & Chiu, 2019). Effective intervention might involve connecting clients with unions, mutual aid networks or community organizations that address structural sources of distress, alongside individual support. This does not mean abandoning clinical care—it means expanding our understanding of what healing requires and resisting capitalism's tendency to pathologize normal responses to abnormal conditions (Adams et al., 2019; Bettache et al., 2020).

Perhaps most fundamentally, mental health advocates should recognize that policies reducing economic insecurity—universal healthcare, stable employment, affordable housing—are themselves mental health interventions, enabling people to make life decisions based on wellbeing rather than survival desperation.

## MAKING THE INVISIBLE VISIBLE: A NEW SYNTHESIS FOR PSYCHOLOGY

These practical interventions—from restructuring classrooms to reimagining clinical practice—share a common foundation: they require psychology to acknowledge capitalism not as background noise but as a primary architect of the phenomena we study. This represents more than adding another variable to our models. It demands recognizing that the ‘culture’ we have long studied through the lens of geography, history and tradition is actively shaped by contemporary economic structures. The individualism we measure, the materialism we document, the competitive anxieties we treat—these are not simply inherited from ancient civilizations or determined by subsistence ecology. They are living responses to present‐day political‐economic conditions, which means they remain open to change. How these patterns manifest depends on the extent to which and how capitalist principles are infused into a society's institutions.

By rendering capitalism invisible, psychology has inadvertently legitimized its psychological costs as natural or inevitable. This is not about rejecting all aspects of market economies or claiming that capitalism explains everything about human psychology. It is about recognizing that economic structures shape culture and consciousness in profound ways that psychology has systematically overlooked. When we study rising anxiety, social isolation, educational stress and workplace burnout, we are studying capitalism's psychological manifestations—whether we acknowledge it or not.

This does not mean treating capitalism as monolithic. Different societies institutionalize capitalist principles with varying degrees of intensity and constraint, producing meaningfully different psychological outcomes. As noted earlier, the same Confucian heritage yields markedly different competitive orientations depending on whether capitalism is regulated or laissez‐faire—suggesting that economic policy may be a more powerful predictor of psychological outcomes than deep cultural traditions. Future research should examine how the syndromes described here manifest under different forms of capitalism (Goudarzi & García Ferrés, 2025)—for instance, welfare capitalism with its stronger worker protections and more collectivized ownership compared to neoliberal or finance capitalism characterized by precarity and hyper‐individualism.

A skeptic might reasonably ask: does capitalism generate individualism, or did pre‐existing individualist values create fertile ground for capitalism's emergence? While the historical origins of this relationship remain open to debate, the contemporary evidence points decisively toward capitalism as the active, maintaining force. Consider the temporal patterns: Japan's marked shift toward individualism over recent decades (Hamamura, 2012) tracks precisely with neoliberal economic reforms that intensified market competition and dismantled lifetime employment protections—not with any revival of ancient cultural traditions (Taniguchi & Kaufman, 2019). China's psychological movement toward greater individualism (Steele & Lynch, 2013) accelerated following market liberalization under Deng Xiaoping, not during the Cultural Revolution's assault on traditional values. Kenya's educated classes exhibit Western‐style independent self‐concepts (Ma & Schoeneman, 1997) not because they rediscovered ancient Greek philosophy, but because higher education serves as a gateway to participation in global capitalist networks. These patterns suggest that whatever historical relationship existed between individualism and capitalism's origins, I argue it is capitalism that now actively produces and reproduces individualist psychology. Cultural traditions do not spontaneously intensify across decades; economic structures that reward individual competition, accumulation and self‐optimization do. The question for contemporary psychology is not which came first, but which force currently shapes psychological experience—and here the evidence implicates capitalism as the generative mechanism maintaining and deepening the individualist orientation.

The question facing psychology is whether we will continue rendering these connections invisible, or whether we will embrace a more complete account of the forces shaping human experience. The latter path requires courage to question foundational assumptions and openness to perspectives long dismissed as ‘too political’. But it also promises a psychology that genuinely serves human flourishing rather than inadvertently legitimizing the systems that undermine it. In an era of mounting mental health crises, social fragmentation and inequality, psychology can no longer afford to ignore the economic structures generating these conditions. Making capitalism visible is the first step toward imagining and building alternatives.

## AUTHOR CONTRIBUTIONS

**Karim Bettache:** Conceptualization; investigation; writing – original draft; writing – review and editing.

## CONFLICT OF INTEREST STATEMENT

The author declares that there is no conflict of interest.

## Footnotes

^1^
Others have argued that psychology does not merely render capitalism invisible but actively promotes it—through historical entanglements with military and surveillance institutions, organizational psychology's service to management interests, and the discipline's reluctance to address its own political commitments (Roberts, 2015). This strong critique has merit; the discipline's complicity runs deep. The present article focuses on invisibility rather than active promotion not because the latter does not occur, but because invisibility may ultimately prove more insidious—precisely because it operates without conscious awareness and thus evades critique.
